# Supplementary material for: Causal Relationship between Aging and Anorexia Nervosa: A White-Matter-Microstructure-Mediated Mendelian Randomization Analysis
Source: Biomedicines. 2024 Aug 16;12(8):1874. doi: 10.3390/biomedicines12081874 (PMC11351342; doi:10.3390/biomedicines12081874)
Supplement: Supplementary file 1 [file biomedicines-12-01874-s001.zip › Supplementary Figure/Figure S4/Singe SNP plot of MR analysis for TL to ubm-b-1961.pdf]

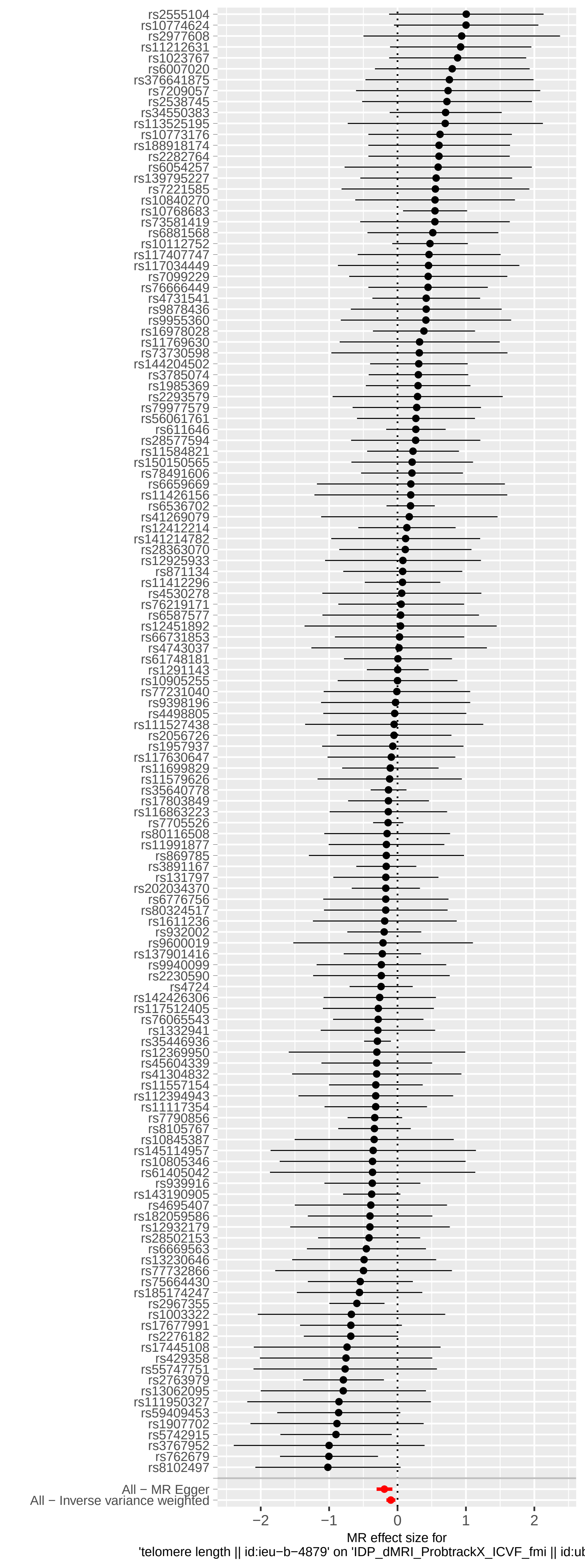
rs2555104
rs10774624
rs2977608
rs11212631
rs1023767
rs6007020
rs376641875
rs7209057
rs2538745
rs34550383
rs113525195
rs10773176
rs188918174
rs2282764
rs6054257
rs139795227
rs7221585
rs10840270
rs10768683
rs73581419
rs6881568
rs10112752
rs117407747
rs117034449
rs7099229
rs76666449
rs4731541
rs9878436
rs9955360
rs16978028
rs11769630
rs73730598
rs144204502
rs3785074
rs1985369
rs2293579
rs79977579
rs56061761
rs611646
rs28577594
rs11584821
rs150150565
rs78491606
rs6659669
rs11426156
rs6536702
rs41269079
rs12412214
rs141214782
rs28363070
rs12925933
rs871134
rs11412296
rs4530278
rs76219171
rs6587577
rs12451892
rs66731853
rs4743037
rs61748181
rs1291143
rs10905255
rs77231040
rs9398196
rs4498805
rs111527438
rs2056726
rs1957937
rs117630647
rs11699829
rs11579626
rs35640778
rs17803849
rs116863223
rs7705526
rs80116508
rs11991877
rs869785
rs3891167
rs131797
rs202034370
rs6776756
rs80324517
rs1611236
rs932002
rs9600019
rs137901416
rs9940099
rs2230590
rs4724
rs142426306
rs117512405
rs76065543
rs1332941
rs35446936
rs12369950
rs45604339
rs41304832
rs11557154
rs112394943
rs11117354
rs7790856
rs8105767
rs10845387
rs145114957
rs10805346
rs61405042
rs939916
rs143190905
rs4695407
rs182059586
rs12932179
rs28502153
rs6669563
rs13230646
rs77732866
rs75664430
rs185174247
rs2967355
rs1003322
rs17677991
rs2276182
rs17445108
rs429358
rs55747751
rs2763979
rs13062095
rs111950327
rs59409453
rs1907702
rs5742915
rs3767952
rs762679
rs8102497
All − MR Egger
All − Inverse variance weighted
−2
−1
0
1
2
MR effect size for
'telomere length || id:ieu−b−4879' on 'IDP_dMRI_ProbtrackX_ICVF_fmi || id:ub
